# The Knife in Uterine Surgery

**Published:** 1888-07

**Authors:** W. E. B. Davis

**Affiliations:** Birmingham, Ala.


### THE KNIFE IN UTERINE SURGERY—ITS USE AND ABUSE.

BY W. E. B. DAVIS, M. D., BIRMINGHAM, ALA.
Delivered in the Omnibus Discussion of the Alabama Medical Association, April 13th, 1888.

*Mr. President and Gentlemen:*

The longer I study gynecology the more thoroughly am I convinced of the too frequent use of the knife in uterine surgery. But because it has been so misused by some as to have been barren of good results, and in no small proportion of cases prolific of bad, is no argument against its proper use.

No one who has witnessed the benefits of trachelorrhaphy can question the efficacy of this operation. Its results have been a boon to suffering women beyond the most sanguine expectations of even Dr. Emmet himself, who first performed the operation. Dr. Goodell finds that one in six of all women who consult him for uterine trouble has ununited lacerations of the cervix, and Dr. Emmet deems it the cause of most uterine disorders. Hence, with so large a per cent. of sufferers as the result of this injury, from subinvolution of the uterus, menorrhagia, leucorrhœa, chronic ovaritis, prolapse of the uterus, and from epithelial cancer, which is claimed by many to be caused most frequently by the acrid discharges resulting from the injury, there is no surprise at Dr. Emmet's operation having attracted so much attention and won for him additional honors from the entire medical profession. Having originated from so great an authority as Dr. Emmet, and having received the endorsement of the greatest gynecologists of the world, it has become unusually popular, and, like all good operations, has fallen into the hands of many who, from ignorance or want of skill, have failed to get satisfactory results, and hence condemn the procedure as not only of no benefit, but dangerous. In addition to the relief afforded for the above affections, sterility, which so often follows a rent in the cervix, is thus removed. There are ununited rents in the cervix which cause no symptoms, and hence require no operation; but it must be remembered that these are exceptional cases.

For the relief of dysmenorrhœa and sterility in some forms of anteflexion of the uterus, division of the cervix backward has gained great results in the hands of many, but is greatly opposed by others. It was performed years ago by many eminent gynecologists, and foremost among them, by our own renowned Sims and by Sir James Y. Simpson, of England, with the most satisfactory results, but in the hands of ignorant operators it has done much harm. Its success depends largely on the after-treatment, and if this is not properly carried out there will often arise very grave symptoms, and if the canal is not kept sufficiently open no benefit will result. It is also for the want of the knowledge necessary for the proper selection of cases that the operation has been so often performed with harm to the patient and caused disappointment in not giving the expected results. Dr. Wilson, of Baltimore, in a paper read before the American Gynecological Society, has made a splendid classification of the indications for its performance. He recommends the operation especially in the following cases:

"1. Those of anteflexion of the uterus, with an elongated, indurated cervix, where the body is bent upon the neck, or the neck upon the body, or where they are bent upon each other, thus forming a more or less acute angle at the internal os.

"2. Those cases of not such acute flexion, but where the cervix is hyperplastic and indurated, as blue as a mulberry and as dense as a cartilage.

"3. Those cases where there is a hard and unyielding band encircling and constricting the internal os, through which the probe passes with difficulty, and gives to the hand the sensation as if passing over rough and dense cartilage, while the finger of the other hand, in the sulcus between the body and neck in front, gains the impression of a strong cord tied around the uterus at the point of union between body and neck."

In the cases mentioned, no other treatment can possibly give so satisfactory results as the cutting operation. It would be unwise to use forcible dilatation with steel instruments, for we would be liable to tear the indurated tissue and it would be as liable to give way in one direction as another, and, too, we would fail to get the results of the cutting operation; for the indurated tissue, even though it did not tear, would soon contract to almost its original condition, and the canal would be as effectually closed as before. The bilateral division of the cervix should not be performed; for, in addition to its failure to relieve the constriction of the canal, we often have the same injury as results from lacerations at child-birth. In the posterior division of the cervix, the circular fibres are paralyzed and the longitudinal still contract; and thus the body and cervix are drawn backwards and the flexion is in a measure corrected, the canal opened, dysmenorrhœa relieved and many barren women made fruitful. I prefer Dr. Wilson's method of performing the operation and have found it free from danger.

The anterior lip of the cervix should be divided for retroflexions with the indications mentioned for anteflexions. This, however, is not required so often as the former operation.

The bilateral division of the cervical canal by Simpson, which is performed for constriction of the canal and the consequences thereof, dysmenorrhœa and sterility, I believe, should never be done, for reasons already mentioned. Peaslee's operation might be performed in such cases where the cervix is indurated, and if this should prove inefficient the anterior or posterior section should be made. After the bilateral operation there is always danger of hemorrhage, peritonitis, cellulitis and death, and hence it should not be done when we can get equal results by less dangerous methods. I have always found that in those cases where forcible dilatation was not admissable that the best results could be had by Sims' antero-posterior operation, and, therefore, I think the bilateral division of the cervix uncalled for.

In cases of inflammation of the cervical mucous lining with pent up acrid discharges, when the cervix is indurated, superficial division of the external os should be practiced. It can be performed with a bistoury or Keuchenmeister's scissors, but dilators should be used afterwards.

The bilateral section of the cervix, to arrest the growth of fibroids and consequent hemorrhage, by Barnes, of England, should no longer occupy a place among the recognized uterine operations for reasons which we will give further on.

It is necessary to amputate the cervix in some cases of hypertrophy with prolapse, and in epithelial and schirrus growths when the cervix alone is involved. The entire uterus should be removed by the vagina if the body is involved by malignant growth and the surrounding organs are not. The cervix can best be amputated after the method of Martin, of Berlin, and the entire uterus as practiced by Schrœder. Should the uterus be too long to be removed per vagina, it should be done after the plan of Dr. Freund by abdominal incision.

In conclusion, gentlemen, I desire to consider the surgical treatment of uterine fibroids of the interstitial and sub-peritoneal varieties. As these growths very rarely terminate fatally, it has been a great question with gynecologists as to the advisability of the operation which has been practiced for their relief; for notwithstanding the fact that they cause great inconvenience from size and hemorrhage, it is a very serious question to perform an operation for their relief, which gives a mortality of seldom less than fifty per cent. It has been the earnest desire of the profession for a less dangerous procedure, and thanks to the labor and achievements of Apostoli, possessed with a strong battery, a galvanometer, an intra-uterine platinum sound and a cutaneous electrode of clay, we are now enabled to do a hysterectomy without an anæsthetic, without pain and without danger, and with skill easily acquired, and with as much relief as the dangerous cutting operation. I was greatly surprised that Dr. Jordan, who read a paper on electricity on yesterday, did not refer to intra-uterine chemical galvano-cauterizations and the results of this treatment on fibroid growths. (Here Dr. Davis described Apostoli's apparatus and gave full instructions as to its use.) According to Apostoli, in almost every instance where this treatment has been used, all symptoms have been relieved, the tumor greatly reduced in size and the patient restored to almost perfect health, and in addition to Apostoli's evidence, we have such men as Keith, of Edinburg, and Smith, of Montreal, endorsing the treatment. Keith says: "The treatment introduced by Apostoli must take precedence of all others. ... The success of his treatment is a great fact, and in saying that I accept in *toto animo* his teachings, I do not speak without some experience of his practice."

Dr. Keith used the treatment 1,200 times in 25 cases and found relief in all but one.

With the evidence we have for this treatment, it does not seem that hysterectomy should ever be justified before electrolysis has been used.

I hope soon to prepare an article giving the results of my own experience with the treatment, based upon a number of carefully recorded cases.

